# Nitrous Oxide and Oxygen Analgesia

**Published:** 1912-08-15

**Authors:** E. S. Barber

**Affiliations:** Chicago


### NITROUS OXIDE AND OXYGEN ANALGESIA.

BY E. S. BARBER, D.D.S., CHICAGO.

I rarely prepare a cavity in a vital tooth without using nitrous oxid and oxygen and rarely use arsenic for devitalization as it is such a simple matter, when preparing cavities in the analgesic stage, to shut off the air and put the patient down to anesthesia with a few whiffs of $N^2O$ & O when the pulp can be exposed and removed and any necessary extractions done without the patient's knowledge.

One great advantage I have derived from this method of pulp extirpation is in the absence of after soreness so common where either cocaine or arsenic is used. I don't remember ever giving a devitalization case another treatment on account of soreness, but have always filled at the next sitting and found the teeth comfortable.

There is a noticeable increase in hemorrhage in these cases, sufficient to irrigate the pulp cavity thoroughly and keep it aseptic while operating without the rubber dam. You have time for the patient to become conscious and can clear your field of operations before the hemorrhage ceases and I don't know any better way of keeping the field aseptic. I usually seal in a treatment of Williams's Carbol-Eugenol liquid for a couple of days and invariably fill at the end of twenty-four or forty-eight hours, finding no tenderness whatever in the tooth or bone.

The proper working stage for cavity and crown preparation is known as analgesia; after that comes the second or excitement stage, and then the third stage of anesthesia.

In analgesia the patient must at all times be kept conscious, able to talk, to spit out and to keep his eyes open. He must never go down so far as the excitement or second stage of anesthesia. In other words, correct analgesia is a state of intoxication, during which the patient at no time becomes unconscious. It does not affect the central nervous system, but acts entirely on the sensory nerves.

I have kept a patient under for an hour and fifteen minutes for the preparation of ten teeth. This patient I was unable to control at the analgesic stage, so put her in the anesthetic stage, and because of the difficulty in controlling her I kept her there and finished all the cavity preparations at one sitting.

*Suggestion* plays a large and important part in analgesia. I think a mixture of about five parts *suggestion* and one part

$N^2O$ & O would be about right for painless cavity preparation.

Oftentimes a patient will show excitement because of fear when in reality you are controlling the pain, so I have adopted *positive suggestion* in handling all my cases.

In analgesia the only thing you have to be careful about is not to let the patient get too much anesthetic. They will often try to get more than you want them to have, so you must be able to control what they get. All that is desired is to prevent pain, which can be done while the patient still has control of all his faculties, and by operating in that stage you are relieved of worry about any bad after-effects which might occur under prolonged anesthesia.

My method of procedure is as follows:

I seat the patient with head slightly forward and the chin dropped a little, just enough so the saliva will run forward and can be taken away by the saliva ejector. This is practically the correct extracting position, and I use it because we have to work largely with unskilled assistants who will sometimes let the patient go to sleep while we are busy, and I don't want my patients swallowing saliva or mucus, as that often produces nausea.

Fit the nose inhaler tight enough so it will stay in place without holding with the hands, and at the same time be comfortable. Open both the inhaling and exhaling valves to accustom the patient to the apparatus and to prove to him by a practical demonstration that he can breathe air as well with the nose inhaler in place as without. By taking plenty of time in this demonstration, I secure his confidence, which is the next step. I then commence positive suggestion, such as "I want you to relax just as though you were very tired and wanted to go to sleep." "You are now entirely comfortable, and I am about to do your dental work without any pain or discomfort to you." "You will stay awake and keep your eyes open during the entire operation and will know what is going on, but won't feel any pain." "You are now breathing air and will hardly notice the change when I turn on the $N^2O$ & O (never mention gas). "You are feeling just right to-day for this anesthetic and will find it a pleasant experience." "If you feel sleepy at any time breathe through your mouth and if there is the slightest twinge from the tooth I'm working on, breathe a couple of deep breaths through the nose and it will go away." "I want you to take this anesthetic entirely yourself and only take it to avoid pain." "It wouldn't do any harm if you took a lot of it, but it is easier for me to work if you stay awake."

When the patient is entirely quiet and breathing naturally, and I insist on their breathing just that way, for this all tends to keep them normal, I signal the assistant to turn on the anesthetic. She has previously run some anesthetic through both bags and allowed it to escape to get rid of any stale $N^2O$ & O remaining from a previous operation. It is hard to describe to a beginner the correct amount of anesthetic to have in the bags, and this detail has taken me months to learn, but one can easily arrive at the amount by filling both bags and turning the indicator to "mix," where both bags will empty equally to a point where they appear collapsed. Shut off the valve at this point and take either of the bags in the hand and they will have considerable anesthetic remaining. This amount is usally enough during analgesia, and after the operation is commenced only enough $N^2O$ & O should be allowed to flow from the cylinders to keep the bags at this point. The amount used is so small as to appear infinitesimal, but the results are better than where more is used. In fact, if pressure is used with air valves open, most of the anesthetic is lost in the atmosphere which will at least treble your expense, and should the air valves be closed your patient will get the effects of too much anesthetic, which will produce choking, nausea, etc., and you will get excitement and anesthesia instead of analgesia. With the indicator of the mixing chamber turned to $N^2O$ and a small amount of $N^2O$ flowing from the cylinder, I next close the inhaling air valve found on the nasal inhaler, and instruct the patient to breathe naturally through his nose. Since the mouth is closed he gets nothing except $N^2O$ and usually from six to eight inhalations brings on a tingling sensation in the fingers and toes and throughout the system, somewhat similar to that felt after striking the so-called "crazy-bone." This stage is usually analgesia. With some, I let them strike their teeth together, and if they feel like wood they are all right, or have some bite their fingers and when they do that without pain they are ready. Another test is the point where the perspiration breaks out on the forehead. Some one of these indications will be noticed in each case, though not all will be found in every case. However, in most cases the operating stage has to be determined with the bur, and the safe rule to follow is to give just as little anesthetic as you can to operate painlessly.

When the analgesic stage is reached the inhaling air valve should be opened, a small amount of oxygen allowed to flow from the oxygen cylinder, and the indicator moved to allow a mix of approximately 25 per cent. oxygen with the $N^2O$ the patient is breathing. At the same time, he is instructed to open his mouth and the operation is begun. At this point the saliva injector should be inserted in the mouth. I would advise a metal tube in anesthetic work, for if you should grow careless and let the patient bite on it, no damage will be done. It can readily be seen that he is getting a very small proportion of anesthetic, for he breathes air through both the mouth and nose, probably more air than $N^2O$ & O combined. In fact, in some cases we have to close the inhaling air valve and increase the pressure on the bags, for often, where the mouth is wide open, no breathing at all is done through the nose and consequently no anesthetic inhaled. Some patients require up to 50 per cent. oxygen, and some none whatever, as a very little will produce hilarity and make operating impossible. However, a few moments' experimenting with the indicator, moving it one point at a time, will indicate the proper mixture, and it should then be left alone as much as possible throughout the operation, for nausea is often produced by the fluctuation of the anesthetic. If pain is felt the valve in the $N^2O$ cylinder should be opened slightly, producing a little more pressure on the bag, though I would first try shutting down the air inhaling valve, and suggest to the patient to breathe through the nose. In some rare instances with the valves and mouth open, only an occasional whiff of $N^2O$ need be allowed to enter the bag to keep the patient quiet. In fact, the right amount of pressure in most cases is just enough to bring the anesthetic to the nostrils. Air is all about us, and while it is in contact with the nose, we can stop breathing it if we want to. This anesthetic should be administered on the same general principle as we get air, which more nearly approaches nature's way than any other I've tried. And since trying this method my patients have remarked so many times at the ease of administration as compared to the pressure method formerly employed.

The two extremes to watch out for in analgesia are hilarity on the one hand, meaning too much oxygen, and unconsciousness on the other hand, which if carried too far will result in cyanosis, caused by too much $N^2O$.

I do not use the mouth prop in analgesia, because the first indication of too much anesthetic is a fluttering of the eyelids, which the operator may not see if he is working fast, and next a gradual closure of the jaw, which he is bound to see at once. With a mouth prop in place the patient would be biting hard before this latter could be detected, and would probably be just into the excitement stage, where one would have to stop work for from one to five minutes till the proper operating stage could be resumed.

Besides, with the jaw stretched wide open the patient has no chance to rest and can't swallow, but might get something in the throat. In grinding cavities a large amount of dust is produced, more than I ever imagined while operating under the old method. The reason is that with a patient awake he usually spits out these accumulations at every opportunity, so they cause no embarrassment. But under analgesia the patient is intoxicated, and if you put his head in one position it will usually stay there and he will not even try to spit out unless you tell him to. Besides the accumulation of dust, most people have a considerable formation of mucus in the nose and throat. This is especially noticeable in damp climates and where the patient has a cold. All these accumulations must be removed occasionally or they will produce vomiting, and vomiting has all along been the one drawback which I've found hard to overcome. We have overcome it at last and by following the few simple suggestions outlined above. Under analgesia, the patients spits out these accumulations himself into the small hand cuspidor held by the assistant, (I do not advise letting him spit into the regular chair cuspidor, as that is too much exertion for anyone partly anesthetized. The patient is tired and practically helpless and the operator should recognize this and not put him to any exertion.) Under prolonged anesthesia lasting from ten minutes to several hours, these accumulations must be removed mechanically by using sponges of gauze held in a long curved pair of artery forceps. This is rather difficult for the beginner to do, so it is readily seen that the best results can be obtained by the average practitioner through using the analgesic stage.

Upon noticing either of the sleep indications, I tell the patient to breathe through the mouth, and this is a signal to the assistant to turn off the anesthetic. On the other hand, if I notice the patient begin to wince I tell him to breathe deep through the nose a couple of times, and that sensation will disappear. This is a signal for the assistant to turn on more anesthetic, while the patient believes he has the matter entirely under his own control, and it gives him a great deal of confidence. In fact, at the second sitting I never have any difficulty handling the patients, for they have entire confidence that everything is coming out all right. For this reason, I usually explain the workings of the machine and the anesthetic to the patient and try it on him experimentally at a previous sitting rather than to do it all new at the sitting where I begin preparing cavities. A patient's actions are largely governed *by direct or indirect suggestions* from the operator. If the dentist suggests pain they get ready to be hurt, but if he has confidence in himself and is backed up by $N^2O$ & O, he just simply breathes confidence to the patient from every pore, and the suggestion of perfect confidence has in every case in my practice overcome all the objections a patient could raise.

As to the old idea of using sharp burs to excavate painlessly, that is all folly, for sharp burs hurt and heat up teeth nearly as badly as dull ones. However, I believe in using plenty of sharp burs, usually three or four for each cavity, besides maybe one or two sharp stones. In this way I can work fast, and usually prepare two inlay abutments for bridgework in fifteen to twenty minutes.

I use a stream of cold compressed air under about thirty pounds pressure directed on the bur to keep the bur and cavity from heating. It also drives away the debris and saliva, so I can readily see where I am going and no matter how fast I have worked I have never had any trouble from dead pulps. In using inlay stones I dip them in cold water every few revolutions to prevent overheating.

Following is a list of things which I have found, if carried out carefully, will bring about the most satisfactory results.

1. Your operating room should be clean and quiet and of moderate temperature.

2. It is best that the patient should not eat for four hours before operating under analgesia, and the ideal time to operate is at mealtime.

3. The bowels should be cleared and the bladder empty before giving the anesthetic.

4. All tight clothing, such as corsets, waistbands, neckbands, etc., should be loosened.

5. All operating instruments should be concealed so that the patient will not become unduly excited or nervous.

6. Regardless of his feelings, the dentist must at all times appear calm and collected and entire master of the situation. Any nervousness on his part is certain to cause the patient to feel uneasy.

7. With anaemic patients, the nitrous oxid should be warmed to blood temperature, as it often reaches a temperature of from 10 to 20 degrees below the freezing point after running a considerable time.

8. The dentist should always be prepared for an emergency by having a hypodermic syringe charged with a solution of about 1-30 gr. of strychnine, tongue forceps, mouth gag, brandy and ammonia ready for instant use.

After using for some time the regular $N^2O$ and oxygen apparatuses used by most dental specialists and with which my nurse had been long familiar, I was asked to try the new Clark outfit, and got one of them on suspicion. From the time of the first operation we never made use of the old machines again, because of the extreme safety of this apparatus and the ease with which it can be operated.

When the bags are sufficiently filled and the inhaler is adjusted, the dentist should depend upon his assistant to operate the machine. A simple movement of the hand would mean more oxygen or more $N^2O$ as the case may be. The mixture of the gases is governed altogether by one large handle and the mixture can be arranged to the satisfaction of the dentist by simply turning one valve in one direction or the other, which, of course, all those who have had any experience giving anesthetics of this character will appreciate.

After operating I take away the inhaler and instruct the patient to keep the eyes closed a few minutes and to remain absolutely quiet for a time. I find that pure oxygen has the instantaneous effect of bringing the patient up like a knife thrust and often causes nausea and vomiting whereas, should the patient be allowed to remain quiet and breathe air till recovered he will be ready after analgesia to put on his hat and leave the office in a few minutes.—Abstract of a paper printed in the July *Oral Hygiene.*

